# A temporal integration mechanism enhances frequency selectivity of broadband inputs to inferior colliculus

**DOI:** 10.1371/journal.pbio.2005861

**Published:** 2019-06-24

**Authors:** Chen Chen, Heather L. Read, Monty A. Escabí

**Affiliations:** 1 Electrical and Computer Engineering, University of Connecticut, Storrs, Connecticut, United States of America; 2 Biomedical Engineering, University of Connecticut, Storrs, Connecticut, United States of America; 3 Department of Psychological Sciences, University of Connecticut, Storrs, Connecticut, United States of America; New York University, United States of America

## Abstract

Accurately resolving frequency components in sounds is essential for sound recognition, yet there is little direct evidence for how frequency selectivity is preserved or newly created across auditory structures. We demonstrate that prepotentials (PPs) with physiological properties resembling presynaptic potentials from broadly tuned brainstem inputs can be recorded concurrently with postsynaptic action potentials in inferior colliculus (IC). These putative brainstem inputs (PBIs) are broadly tuned and exhibit delayed and spectrally interleaved excitation and inhibition not present in the simultaneously recorded IC neurons (ICNs). A sharpening of tuning is accomplished locally at the expense of spike-timing precision through nonlinear temporal integration of broadband inputs. A neuron model replicates the finding and demonstrates that temporal integration alone can degrade timing precision while enhancing frequency tuning through interference of spectrally in- and out-of-phase inputs. These findings suggest that, in contrast to current models that require local inhibition, frequency selectivity can be sharpened through temporal integration, thus supporting an alternative computational strategy to quickly refine frequency selectivity.

## Introduction

Unlike the visual and somatosensory modalities, the mammalian auditory system is unique for the vast amount of neural circuitry in the brainstem and midbrain. The extensive amount of divergence and convergence in the ascending auditory pathway allows for considerable transformations of spectral and temporal sound selectivities. However, our understanding of how sound representations are newly constructed or transformed at each stage of the auditory pathway remains largely a matter of inference because only a handful of studies have addressed how sound transformations are mediated on a neuron-to-neuron basis between mutually connected neurons. Recordings from pre- and postsynaptic action potentials in the auditory brainstem indicate that, while subtle changes in selectivity can be observed in the calyx and endbulb of Held synapses, synaptic transmission in these large synapses is relatively reliable so that sound preferences are largely inherited from the presynaptic inputs [1–4]. By comparison, in recordings from connected auditory thalamocortical neuron pairs, individual thalamic inputs contribute only a small fraction of the overall cortical activity, and dramatic changes in spectrotemporal preferences can be constructed de novo [5]. Similar transformations have also been described in the visual system, whereby retinogeniculate projections tend to preserve center-surround selectivities for visual space [6] and geniculate-cortical projections lead to newly constructed orientation selectivity [7]. Within the inferior colliculus (IC), receptive fields are exceptionally diverse, and response preferences could be partly inherited from converging brainstem inputs [8, 9]. On the other hand, the IC’s local anatomic circuitry and extensive brainstem inputs also allow for considerable construction of response preferences, and receptive field can be shaped by local mechanisms [10–12]. Yet direct evidence for how brainstem inputs are transformed on a neuron-to-neuron basis is lacking.

We demonstrate a transformation in spectral selectivity between putative brainstem inputs (PBIs) and recipient IC neurons (ICNs) accomplished by temporal integration of broadly tuned inputs. PBI response waveforms resemble and are consistent with presynaptic potentials, as previously observed in the ascending auditory system [1, 2, 13–15] and the squid giant axon [16]. Two distinct groups of PBIs were identified with different spectrotemporal properties that reflect the presence of functionally distinct inputs to the IC. Neighboring ICNs within a radius of approximately 100 μm had considerably lower temporal precision than the simultaneously recorded PBIs but exhibited a substantial enhancement in spectral selectivity that is comparable to perceptual “critical band” resolution [17, 18]. This transformation is accurately replicated in a neuron model in which enhancement in frequency selectivity does not require local surround inhibition and is instead accomplished by temporal integration of spectrally in- and out-of-phase inputs to the IC.

## Results

We first demonstrate that prepotentials (PPs) can be recorded extracellularly in the IC with physiological properties that are indicative of brainstem input. Second, we show that precisely correlated firing between PBIs and ICNs is organized into frequency bands that are consistent with the layered brainstem input to the IC. Paired PBI and ICN recordings are then used to identify distinct neural sources to IC and to demonstrate that temporal coding precision degrades—while frequency selectivity is substantially enhanced—between PBI sources and neighboring ICNs. Finally, a neuron model constructed from the PBI receptive fields demonstrates that the temporal integration of the incoming inputs can enhance spectral selectivity as observed for the neighboring ICNs.

### Differences between PPs and ICN spikes

**Fig 1:**
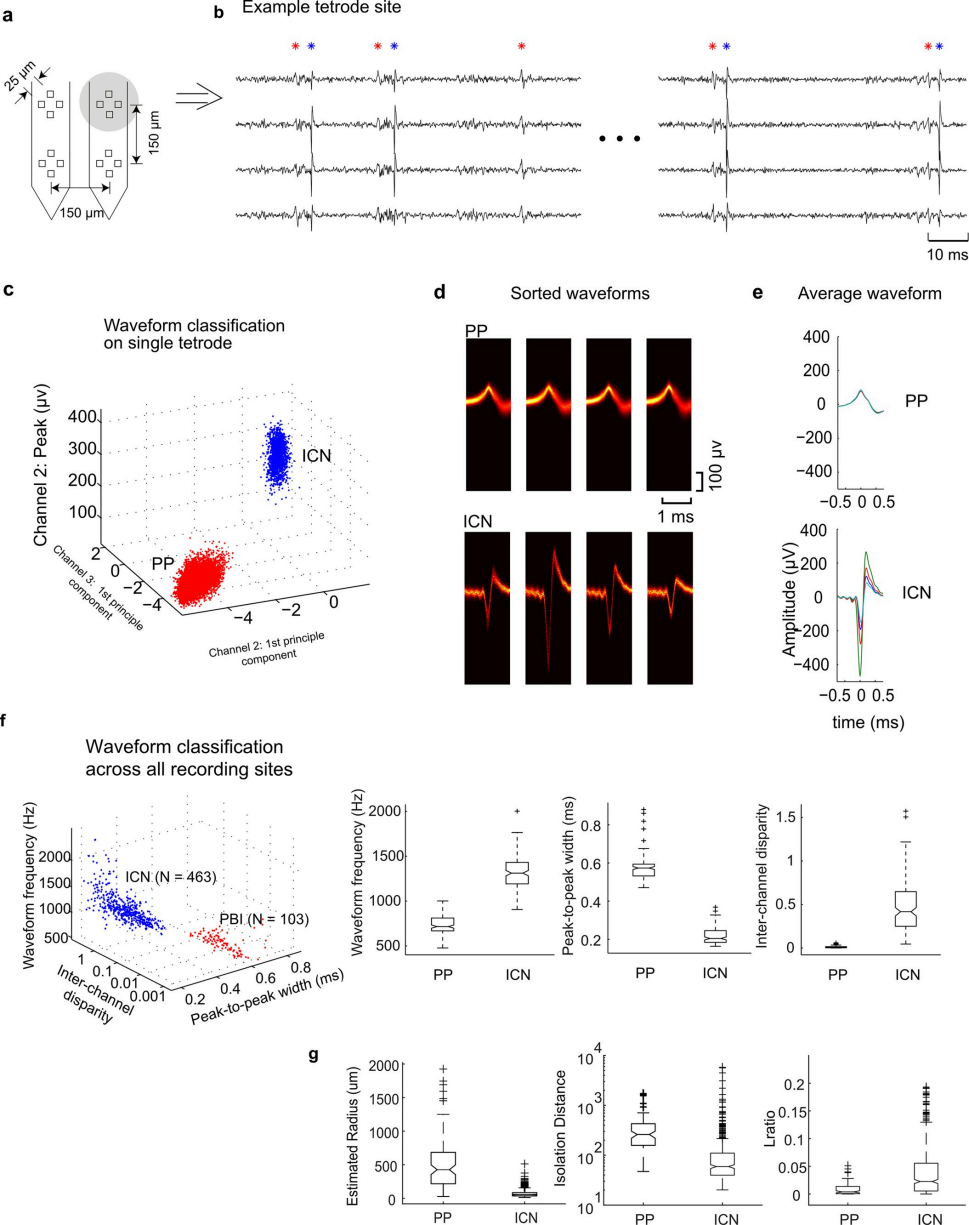
Identification of PPs and ICNs. (a) A tetrode array was used to record and isolate single unit activity and PPs from the IC. (b) Example recording trace showing simultaneously recorded PP and ICN waveforms on 4 tetrode channels. Detected PP events are noted in red (asterisk), whereas detected ICN events are noted in blue (asterisk). Peak amplitudes and first principle components from the 2 groups of events exhibit distinct clusters (c). Waveform density plots (panel d; black = low probability; orange = high probability) and average waveforms (panel e) are shown for each of the 4-tetrode channels. PP waveform durations are longer than the ICNs, and the average waveforms of the PP on the 4 channels are highly consistent in shape and amplitude and thus exhibit low interchannel disparity. PPs and ICNs from all recording sites are identified by performing cluster analysis on the waveform properties (panel f; see Materials and methods; PP versus ICN mean ± SD: waveform frequency, 714 ± 105 versus 1,248 ± 245 Hz; peak-to-peak width, 0.58 ± 0.08 versus 0.27 ± 0.07 ms; interchannel disparity, 0.02 ± 0.07 versus 0.48 ± 0.68). (g) PPs have a larger recording radius (54.8 versus 426.1, median), larger isolation distance (260.8 versus 59.3, median), and lower *L*-ratio (0.004 versus 0.23, median) than the neighboring ICNs (Wilcoxon rank sum, *p* < 0.001). Underlying data can be found in S1 Data and S1 Code. IC, inferior colliculus; ICN, inferior colliculus neuron; PP, prepotential.

Tetrode arrays (Fig 1A) were used to record single neuron activity in the central nucleus of the IC (ICC). Neural recordings were performed to identify well-isolated neighboring single units across the tetrode array while delivering dynamic ripple sounds [19, 20]. Candidate action potentials or “spikes” were detected offline and sorted from each 4-channel recording trace (Fig 1B) using spike sorting analysis applied to the waveform peak amplitudes and first principal components (e.g., Fig 1C; see Materials and methods). Post hoc cluster analysis of the identified waveforms revealed a distinct PP waveform in a subset (19%) of recording sites (e.g., Fig 1F, see Materials and methods). Recordings from one tetrode site (Fig 1B) illustrate the temporal contiguity between an example PP (red asterisks) and an ICN spike (blue asterisks). These PPs (red asterisk) preceded the ICN spike (blue asterisk) although in some instances isolated PPs with no adjacent spike were observed (i.e., see third red asterisk). These waveforms were identified independently and blindly by the spike detection and sorting algorithm, and there was no attempt to manually identify paired spikes and PPs during or after the neural recording.

Several features of the PPs suggest they are not simply due to postsynaptic potentials such as those that are evident in the local field potential (LFP). First, LFP signals are obtained by filtering neural traces below 300 Hz, whereas here, all of the neural signals are filtered above 300 Hz, and very brief waveform snippets of only 1.5 ms are used for spike sorting (see Materials and methods). Second, LFPs have response amplitudes that increase in a graded fashion with sound level [21]; however, peak amplitudes of the PPs were not correlated with the instantaneous sound pressure levels of the dynamic moving ripple (DMR) sounds (PP amplitudes versus instantaneous SPL of the DMR sound: r = 0.067 ± 0.053, mean ± SE). Third, summed postsynaptic potentials in the LFP do not have refractory periods, whereas the PPs have a relatively low detection rate (median = 2.6 spikes/s) and a well-defined interevent refractory period between consecutive events (PP: 1.14 ms–9.2 ms [median 1.9 ms]) as do the ICN spikes (1.14–30.7 ms [median 2.1 ms]). One possibility is that PPs are failed action potentials from one of the recorded ICNs. However, this is unlikely because one would expect a potential associated with a “failed spike” to have the same initial phase and polarity as the waveform of the successful spikes. However, this is not the case because 90% of recording sites had PP waveforms with inverted polarity when compared to simultaneously recorded spikes from ICNs (e.g., Fig 1D and 1E).

Several observations also suggest that PPs and ICN waveforms originate from distinctly different sources. For single recording sites, PP and ICN spike waveforms form well-isolated clusters (Fig 1C, red and blue, respectively). As can be seen in this example and subsequently observed for all the data (Fig 1F), PPs have (1) a power spectrum with an energy maximum at lower frequencies (667 Hz), (2) longer peak-to-peak width (0.57 ms), and (3) a smaller peak-to-peak amplitude (139 μV) than that of the identified ICN spikes (1,335 Hz, 0.2 ms, 735 μV). These differences do not alone rule out the possibility that the PPs originate from a different class of ICN because waveform shapes can vary with morphology and cell size. However, if the PPs are spikes from a different class of neuron, the waveform amplitudes are expected to vary considerably across tetrode channels. For the example ICN spike waveform, the amplitude varies considerably across tetrode channels (e.g., Fig 1D and 1E, bottom row), as is typical of spike recordings in IC [19, 22] and other brain areas [23, 24]. In contrast, the PP waveform amplitudes are homogeneous and do not vary across tetrode channels (e.g., Fig 1D and 1E, top row). For this example recording site, the interchannel disparity (see Materials and methods) of the PP was nearly 2 orders of magnitude smaller than the simultaneously recorded ICN spikes (0.005 versus 0.32, median; Wilcoxon rank sum, *p* < 0.001), indicating that the PP waveforms are substantially less variable and more homogeneous across tetrode channels than the corresponding ICN spikes. Post hoc cluster analysis applied to 3 waveform parameters (peak frequency, peak-to-peak width, interchannel disparity) for all recording sites in this study reveals 2 distinct clusters (see PP and ICN clusters, Fig 1F). Indeed, the PP cluster, on average, has maximum energy at lower frequencies, longer peak-to-peak width, and an interchannel disparity roughly 2 orders of magnitude smaller (median spectrum peak = 715 versus 1,311 Hz, Wilcoxon rank sum, *p* < 0.001; median peak-to-peak width = 0.57 ms versus 0.20 ms, Wilcoxon rank sum, *p* < 0.001; median interchannel disparity = 0.006 versus 0.42, Wilcoxon rank sum, *p* < 0.001).

For both the ICN and PP clusters, we also computed the *L*-ratio and the cluster isolation distance (Fig 1G). Together, these two metrics account for how well separated a cluster is from its neighboring clusters [25]. The PP clusters have a higher isolation distance (260.8 versus 59.3, median; Wilcoxon rank sum, *p* < 0.001) and lower *L*-ratio (0.004 versus 0.023, median; Wilcoxon rank sum, *p* < 0.001) compared to ICN clusters. Together, this indicates that PPs form higher-quality clusters that are more broadly separated from their neighboring clusters. In addition, the PPs are consistently inverted in polarity (90% of recording sites) and have smaller peak-to-peak amplitudes than the ICN spikes (median PP = 94 μV; median ICN = 164 μV; Wilcoxon rank sum, *p* < 0.001). The above results indicate that the PP and ICN spike waveforms are consistently different and nonoverlapping in shape or interchannel disparity and overall form more homogenous and isolated clusters. This rules out the possibility that the PP waveform arises from a nearby ICN that happens to spike with temporal contiguity.

One possibility is that PPs are action potentials from distant neurons to the recording site, which one would expect to have smaller peak-to-peak amplitude as well as temporally elongated and more homogeneous waveforms as a result of volume conduction in the neural tissue. This is unlikely because we have previously estimated the recording radius of our tetrodes to be approximately 100 μm for spiking neurons [19, 26]. Furthermore, as will be described in detail below, physiologic properties also rule out this possibility. The recorded PPs have consistently shorter sound-evoked latencies and higher temporal precision and are matched in best frequency (BF) to neighboring ICNs. If the PPs arose from distant ICNs, there is no a priori reason for latencies to be consistently shorter than those of simultaneously recorded ICNs, and BFs would differ as shown previously [19].

### Possible sources of PP waveforms

The waveform statistics for PP waveforms indicate that these potentials are distinguishable and differ from ICN waveforms in unique ways. One possibility is that PPs correspond to presynaptic activity from ascending brainstem sources such as fiber potentials, presynaptic action potentials, or summed potentials from a single or a few neighboring input fibers. Alternately, PPs may be generated postsynaptically such as for dendritic potentials, somatic excitatory postsynaptic potentials, or failed action potentials.

To better understand the possible source or sources responsible for PP waveforms, we used the tetrode triangulation method to estimate the recording radius for both the ICN and PP waveforms [24]. While the recording radius of an electrode is partly dependent on the electrode properties (e.g., impedance, contact size, etc.), it is also partly dependent on the source properties such as the source spatial geometry and the source voltage amplitude [24, 27]. Thus, if ICN and PP waveforms originate from different and distinct anatomical sources, the estimated recording radius for each is expected to be different. Indeed, estimates of the recording radius indicate that ICNs arise from a relative nearby source to the tetrode with an estimated recording radius of <100 μm (median 54.8; range = 13.4–512.5 μm; Fig 1G), comparable to our previous estimates [19]. In contrast, the estimated recording radius of PPs is significantly larger (median 426.1; range = 26.7–1925 μm; *p* < 0.001, Wilcoxon rank sum test), indicating that PP either arises from a distant point source (e.g., distant neuron) or, alternately, from a relatively larger anatomical source. We argue that the former possibility is unlikely because, as we outline in detail in subsequent sections, PP sound response properties are distinctly different from ICNs, and PPs are matched in BF to the neighboring neurons (most sites within approximately 1/3 octave), which is not expected strictly for distant neurons or sources. The difference in recording radius also provides support against the possibility that PPs are failed action potentials because, otherwise, their estimated recording radius would be similar to that of ICNs.

Another clue indicating that the PPs are distinctly different and likely originate from sources different from ICN spikes is that they had a consistently inverted polarity (90% of sites) relative to the recorded ICN spikes. It is possible that PPs are summed afferent fiber potentials onto dendritic terminals of ICN, as modeling and experimental studies have shown that potentials near the dendritic terminals can be inverted, temporally wider, smaller in amplitude, and less variable across spatial locations (as for the PPs) than those recorded near the soma [27]. PPs with inverted polarity to the neighboring spikes have also been observed for presynaptic action potentials observed in other auditory structures and squid giant axon [14–16]. Although such PPs have been previously identified only in auditory brainstem neurons with large calyx synapses, calyx-like inputs have been recently identified in IC [28] that could theoretically produce similar potentials.

The waveform statistics and physiological properties support the hypothesis that PPs arise from a presynaptic source and are unlikely to arise from postsynaptic sources (failed action potentials, synaptic activity/LFP, or ICN action potentials). Although our data cannot rule out the possibility that PPs are summed potentials from multiple inputs to IC, their low firing rates, clearly identified refractory period, acoustic response properties, and their temporal relationship to ICN (described below) suggest that PPs may originate from one or a few brainstem inputs. For this reason, we refer to them as PBIs with the caution that they may in fact represent a handful of neuron inputs to a local IC neighborhood.

### Transformation of frequency preferences and precisely correlated firing

**Fig 2:**
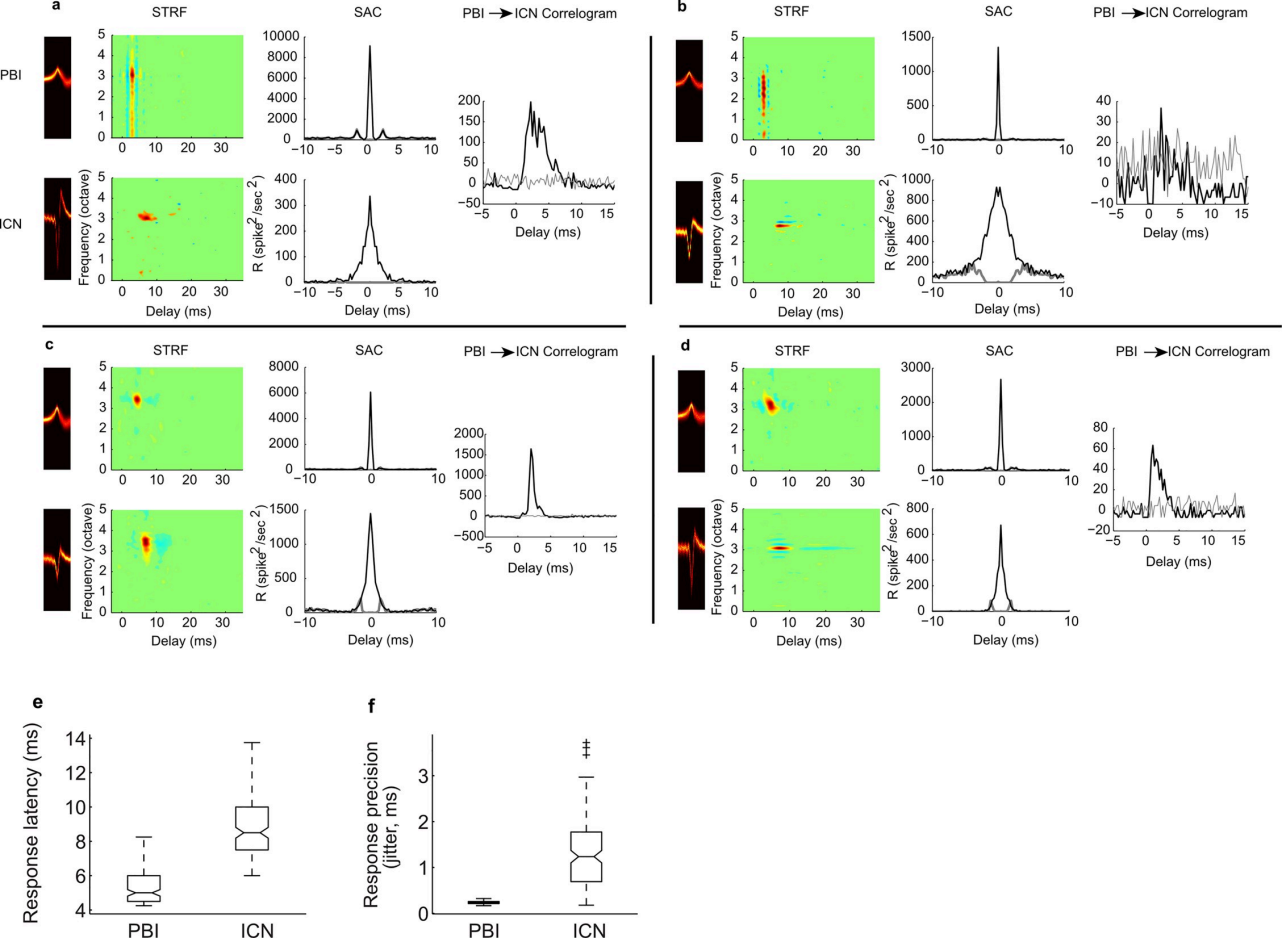
Spiking and spectrotemporal integration characteristics of PBI-ICN pairs recorded from the same tetrode. In each example, we show the STRFs (left panels, top row for PBI and bottom row for ICN), the spike train SACs (middle panels), and the crosscorrelogram between PBI and ICN (right panel). Only statistically significant samples of the STRF (*p* < 0.001) are shown, and samples that do not exceed the significance threshold are set to zero (same convention for all figures; red indicates excitatory response above the baseline firing rate, blue indicates suppressive and/or inhibitory response, and green is the baseline activity). The units of frequency are in octave relative to 1 kHz. As seen in these 4 examples, PBIs often have broader bandwidths than ICNs (panels a, b, d). PBIs also exhibit shorter response latency and faster integration times (panels a–d). Finally, spike-timing precision is higher for PBIs as seen in the SAC (panels a–d, gray line corresponds to the unshuffled autocorrelogram with central peak removed). Significant correlated activity was observed in the PBI-ICN crosscorrelogram between 0 to 5 ms delay in 89% of PBI-ICN pairs (*t* test, *p* < 0.001, gray line corresponds to the interspike interval shuffled crosscorrelogram). Overall, PBIs have shorter response latencies (panel e; range 4.2–8.2 ms; median 4.9 ms) and higher temporal precision (panel f; spike-timing jitter: range 0.17–0.32 ms; 0.23 ms median) compared to that of ICNs (latency range 6–13.8 ms; 8.5 ms median; jitter range 0.2–3.7 ms; 1.2 ms median, respectively). Underlying data can be found in S2 Data and S2 Code. ICN, inferior colliculus neuron; PBI, putative brainstem input; SAC, shuffled autocorrelogram; STRF, spectrotemporal receptive field.

Next, we characterized the functional differences between neighboring PBIs and ICNs. We first compared the spectrotemporal properties between paired PBIs and ICNs that are obtained from the same recording location. At any given recording location, there are 4 tetrodes (4 × 4 electrodes; Fig 1A) each separated by 150 μm. We identified 37 PBI-ICN pairs within the same tetrode and 121 pairs existing within adjacent tetrodes. Fig 2 shows example spectrotemporal receptive fields (STRFs; left panels) and spike train correlograms from 4 same-tetrode PBI-ICN pairs. Several key differences can be observed between PBI and ICN STRFs. First, PBIs always have shorter response latencies than the paired ICNs (Fig 2A–2D, PBI = 4.3, 4.5, 6.0, and 6.1 ms; ICN = 8.2, 10.0, 8.9, and 10.0 ms), which is consistent with the hypothesis that PBIs represent the spike trains from input sources to the IC. Secondly, PBIs have shorter integration times than the paired ICNs (a–d, PBI = 1.7, 1.6, 2.8, and 3.0 ms; ICN = 4.6, 5.4, 3.6, and 7.3 ms) as indicated from the duration of the STRF. Furthermore, PBIs generally have broader spectral bandwidth than the corresponding ICNs (a–d, PBI = 2.3, 1.5, 0.50, and 0.60 octave; ICN = 0.34, 0.17, 1.0, and 0.18 octave). Finally, sideband inhibition tended to be more narrowly tuned and more pronounced for ICNs as in b and d. These general properties were not exclusive to sites containing PBI because there were no significant differences in response latencies, bandwidths, or integration times for ICNs at sites that did not contain PBI (paired *t* test, *p* = 0.11, 0.87, and 0.11, respectively). Overall, these examples demonstrate that spectrotemporal preferences are substantially different between PBIs and ICNs.

Statistics of the sound-evoked response properties further support the hypothesis that the recorded PPs arise from input sources other than IC. First, PBIs have shorter response latencies (Fig 2E, range 4.2–8.2 ms; median 4.9 ms) compared to ICNs (latency range 6–13.8 ms, median 8.5 ms). Although PPs and ICN spike waveforms are identified blindly and independently solely based on waveform characteristics using cluster analysis, they always exhibited shorter sound-evoked latencies than ICNs found on the same tetrode (100% of pairs), which is consistent with arising from an input source. Also, as seen in the example shuffled autocorrelograms (SACs), spike-timing precision differed substantially between PBIs and ICNs (Fig 2; a–d, PBI = 0.30, 0.19, 0.23, 0.23 ms jitter; ICN = 1.27, 2.23, 0.79, 0.69 ms jitter). Overall, PBIs have substantially higher temporal precision (Fig 2F, spike-timing jitter: range 0.17–0.32 ms; 0.23 ms median) compared to that of ICNs (latency range 6–13.8 ms, 8.5 ms median; jitter range 0.2–3.7 ms, 1.2 ms median, respectively).

**Fig 3:**
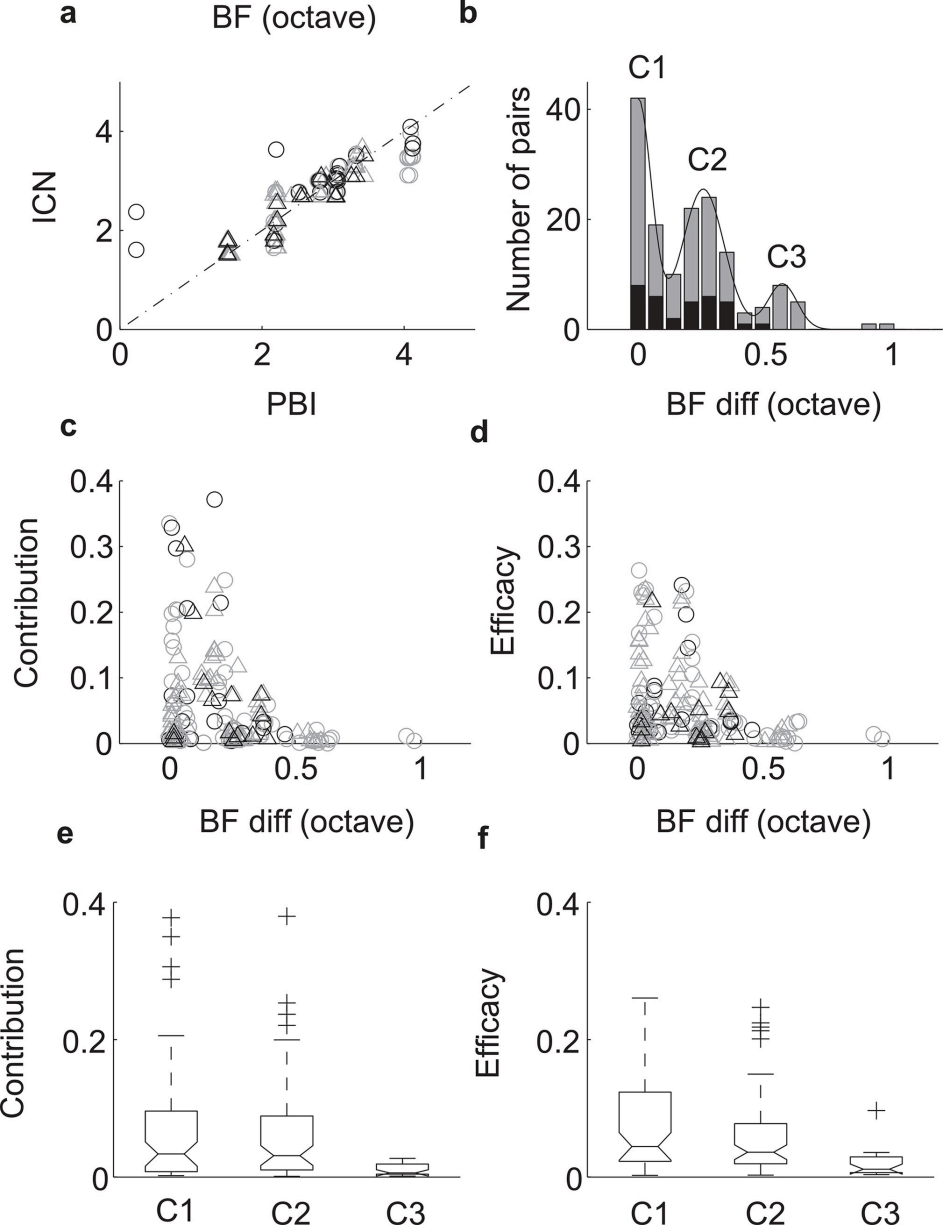
BF clustering and correlation strength are consistent with frequency-band laminar inputs to IC. (a) BFs are closely matched for PBI-ICN pairs recorded on the same tetrode (black; *n* = 37) or adjacent tetrodes (gray; *n* = 121). Open circle denotes broadband PBI, and triangle denotes narrowband PBI. (b) The BF difference between simultaneously recorded PBI-ICN pairs cluster at intervals of 0, 0.26, and 0.56 octave (3 points out of range not shown; cluster 1–3 = c1 − c3; 3 samples >1 octave not shown; black = same tetrode; gray = adjacent tetrodes). The frequency separation between adjacent peaks in the histogram (approximately 0.28 octave) is consistent with the frequency-band spacing reported previously in the cat [32] and is well predicted by a sum of Gaussian model (chi-squared goodness of fit test, χ^2^ = 0.92, *p* = 0.63, df = 2). The contribution (panels c, e) and efficacy (panels d, f) both decrease with increasing BF difference. For the same tetrode pairs (black), the strongest contribution and efficacy are observed for BF difference of zero octave (cluster 1 > cluster 2 and 3; Wilcoxon rank sum, *p* < 0.001). Underlying data can be found in S3 Data and S3 Code. BF, best frequency; C1, cluster 1; C2, cluster 2; C3, cluster 3; IC, inferior colliculus; ICN, IC neuron; PBI, putative brainstem input.

Many of the identified PBI-ICN pairs also exhibited delayed but precisely correlated firing as would be expected for an input neuron source. The crosscorrelograms (Fig 2) between paired PBIs and ICNs reveals a significant delayed peak (0–5 ms; 2.75 ms median; *p* < 0.001, bootstrap *t* test) for 78% of pairs (same tetrode = 89%; adjacent tetrodes = 75%; 1.1 ms median correlation width). Thus, PBIs responded with shorter sound-evoked latencies that were temporally synchronized with the simultaneously recorded ICN, indicating a tight temporal relationship between PPs and ICN pairs and consistent with the hypothesis that PBIs are an input to IC. Had PPs waveforms originated from failed action potentials, we would not expect a strong correlation between paired PPs and ICN spiking. Also, this is inconsistent with the possibility that PBIs are a distant ICN because there is no a priori reason for such a group to consistently have shorter sound-evoked latencies and similar frequency selectivity (Figs 2E and 3A). Finally, such precisely correlated firing is not expected between distant ICNs [26].

The relatively long latency between paired PBIs and ICNs (2.75 ms median) is intriguing in light of the fact that the PPs in brainstem auditory structures precede postsynaptic spiking activity typically by less than 1 ms [1, 2]. If PBI contributes to the postsynaptic activity of ICNs, one possible explanation for this long latency is that the cell membrane integration time constants—which tend to be longer in the midbrain—contribute to the longer measured latencies. We tested this by simulating an integrate-and-fire neuron that received Poisson spiking activity as input using parameters that account for IC activity [26, 29]. When we used a time constant comparable to the average for IC (5 ms) [30], the measured correlation peak delay was 2.4 ms, which is comparable to the median measured value between PBIs and ICNs. By comparison, latencies approached 1 ms when a 1 ms time constant was used for the simulation. Thus, the relatively long latency values observed in the PBI-ICN correlograms can be accounted for by temporal integration of the cell membrane.

All of the PBI-ICN pairs identified from the same tetrode were closely matched in BF with the exception of 3 pairs (within 0.5 octave; Fig 3A). When we examined the distribution of BF difference between paired PBIs and ICNs, 3 distinct clusters (Fig 3B, c1 = 0–0.12 octave; c2 = 0.12–0.45 octave; c3 > 0.45 octave; 3 samples >1 octave not shown; octave frequency referenced relative to 1 kHz) were observed that are indicative of the IC anatomical frequency-band lamina organization [31, 32]. This clustering was accurately accounted for by a mixture of Gaussian model with peaks at intervals corresponding to the reported interlaminar BF separation (chi-squared goodness of fit test, χ^2^ = 0.92, *p* = 0.63, df = 2). BF differences for same-tetrode PBI-ICN pairs were clustered about 0 and 0.26 octave (Fig 3B, black). The measured BF difference for the second cluster closely matches the previously reported laminar separation of 0.28 octave in the cat [32]. When we compared BF differences for PBI-ICN pairs recorded on adjacent tetrodes, a third cluster was observed at 0.56-octave BF difference (Fig 3B, gray). Given that the anatomical lamina spacing in cat is roughly 150 μm [33, 34] and that the estimated recording radius of ICNs on the same tetrode and adjacent tetrodes are approximately 100 and 185 μm, respectively [19], for ICNs, the observed clustering of BF differences is consistent with the hypothesis that PBI-ICN pairs in a recording site were mostly within a single lamina and occasionally spanned 2 (first and second cluster [C1 and C2] observed for same tetrode and adjacent tetrodes) or 3 (third cluster [C3] seen for adjacent tetrodes only, Fig 3B gray) as expected from IC anatomy. This highly structured frequency organization between PBIs and ICNs supports our hypothesis that PBIs do not correspond to distant ICNs because BF differences would otherwise be much larger and would not form distinct clusters at approximately 0.3 octave separation. Instead, they are consistent with a laminar input to IC.

For each pair, we measured how effective PBIs are at eliciting ICN spikes (efficacy: 0.07 ± 0.06, mean ± SE) and how much each PBI contributes to the ICN response (contribution: 0.07 ± 0.08, mean ± SE). Both the efficacy and contribution decrease with the BF difference (Fig 3C and 3D) for each of the paired PBI-ICN frequency clusters (Fig 3E and 3F; c1-c3; Wilcoxon rank sum, *p* < 0.05). This indicates that PBI-ICN pairs that were closely matched in BF—and presumably within the same frequency lamina—were more effective at driving action potentials and contributed more to the ICN responses. Again, these trends support the idea that PBIs are not distant neurons and need to be closely matched in BF to the simultaneously recorded ICN to exhibit correlated firing.

### Functionally distinct inputs from different sources

**Fig 4:**
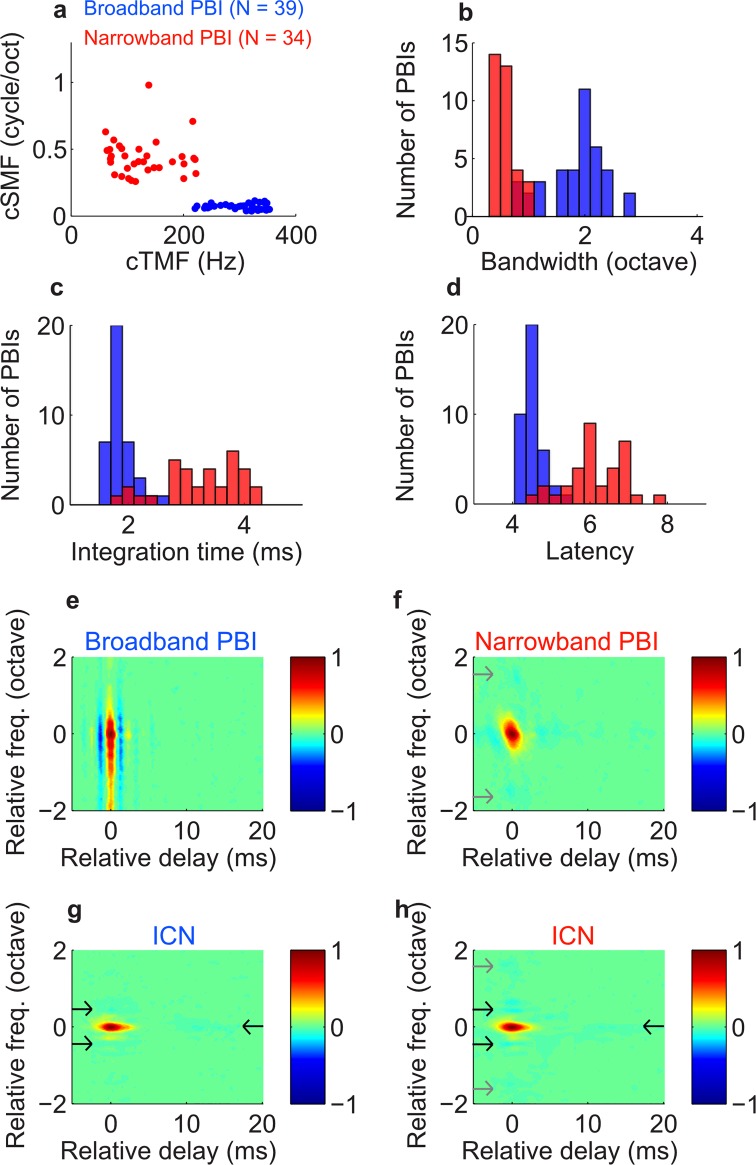
Functionally distinct groups of PBIs have unique spectrotemporal preferences. (a) Cluster analysis on the spectrotemporal modulation parameters (cTMF and cSMF) of the PBIs reveals 2 groups: one with faster temporal modulation preference (blue) and one with finer spectral modulation preference (red). The blue group has broader bandwidth than the red cluster (panel b), and we thus refer to the 2 groups as broadband PBI and narrowband PBI, respectively. Broadband PBI have shorter integration time (panel c) and shorter response latency (panel d) than narrowband PBI. The population-average STRF (normalized and aligned to the STRF peak) is shown for the 2 groups of PBIs (panel e = broadband PBI; panel f = narrowband PBI). The average STRFs for the corresponding paired ICNs are shown for sites with broadband PBI (panel g) and narrowband PBI (panel h). In general, the average ICN has longer integration time, substantially narrower bandwidth, and newly constructed inhibitory regions (panels g and h, black arrows) than the paired PBIs. Underlying data can be found in S4 Data and S4 Code. cSMF, characteristic spectral modulation frequency; cTMF, characteristic temporal modulation frequency; ICN, inferior colliculus neuron; PBI, putative brainstem input; STRF, spectrotemporal receptive field.

If PBIs are generated by multiple anatomic and/or physiologic input sources, it is expected that PBIs might cluster into functionally distinct groups. We found that 53% of PBIs had STRFs with broad frequency tuning (e.g., Fig 2A and 2B; bandwidth 2.3 octave and 1.5 octave) and fast integration times (1.7 ms and 1.6 ms), whereas 47% PBIs had narrower frequency tuning (e.g., as in Fig 2C and 2D, 0.5 octave and 0.6 octave) and slower integration times (2.8 ms and 3.0 ms). We applied a post hoc cluster analysis (Euclidean distance minimum variance algorithm) on the characteristic temporal (cTMF) and the spectral (cSMF) modulation frequency parameters of the PBIs and identified 2 unique response clusters (Fig 4A). The blue cluster prefers high temporal (cTMF, median 309 versus 117 Hz; Wilcoxon rank-sum test, *p* < 0.001) and low spectral (Fig 4A; cSMF, median 0.07 versus 0.42 cycles/octave; Wilcoxon rank-sum test, *p* < 0.001) modulations. This cluster has broader bandwidths (Fig 4B; median 2.0 versus 0.5 octave; Wilcoxon rank-sum test, *p* < 0.001) and exhibits shorter integration times (Fig 4C; median 1.8 versus 3.4 ms; Wilcoxon rank-sum test, *p* < 0.001). We thus refer to the blue cluster as the broadband PBI and the red cluster as the narrowband PBI. Finally, broadband PBIs exhibit shorter latency than the narrowband PBIs (Fig 4D; median 4.5 versus 6.1 ms; Wilcoxon rank-sum test, *p* < 0.001), indicating that they are likely from different pathways or different cell types. These findings are consistent with and indicative of 2 functionally distinct PBI response types.

If PBIs are input to ICNs, differences in spectral and temporal selectivity between the two could, in theory, be shaped by response excitation, inhibition, or both. Averaged STRFs quantify and illustrate the average differences in excitation and inhibition and/or suppression among the response types (Fig 4E for broadband PBI and panel f for narrowband PBI). The average tuning properties of ICNs were substantially different from the corresponding paired PBIs (Fig 4 panels g and h). Furthermore, despite the distinct differences in the PBI receptive fields, ICNs from locations with paired broadband and narrowband PBIs were relatively similar across the two groups and exhibited both narrow bandwidths and similar integration times (bandwidth 0.32 and 0.31 octave; integration time 5.0 and 5.1 ms, respectively). Despite this general similarity, ICNs at narrowband sites exhibited higher cSMF values (1.02 ± 0.04 versus 1.23 ± 0.06, mean ± SE; *p* = 0.006), which indicates selectivity to fine detailed spectral fluctuations and which has been shown to correlate with the amount of sideband inhibition [35]. Indeed, although weak, significant inhibition was observed in the ICN STRFs that matched that of neighboring narrowband PBI (Fig 4H, gray arrows; significant inhibitory peaks are present approximately 1.5 octave in both panel f and panel h). Such inhibition was not observed for broadband sites (Fig 4G). This is consistent with the possibility that some of the ICN receptive field preferences are inherited from PBI sources [5, 8]. However, other inhibitory features seen in ICN STRFs were not present in the paired PBIs (e.g., inhibitory peaks adjacent to the BF; panels g and h, approximately 0.44 and 0.66 octave away; also, long-lasting temporal inhibition; black arrows). This implies that such inhibitory features might be newly constructed in the IC.

### Transformation of temporal and spectral selectivity between neighboring PBIs and ICNs

**Fig 5:**
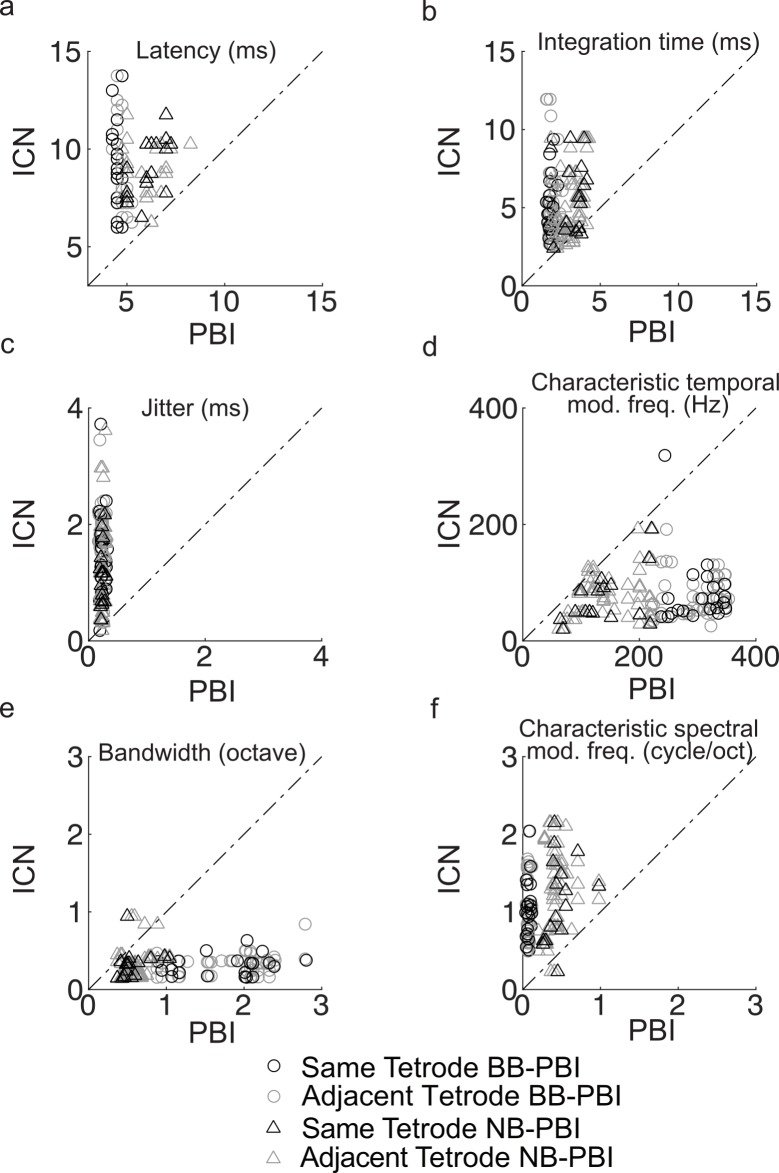
Temporal resolution degrades and spectral resolution is enhanced for ICNs. PBIs exhibit shorter response latency (panel a, 4.9 versus 8.6 ms), shorter integration time (panel b, 2.3 versus 4.3 ms), substantially higher spike-timing precision (panel c, jitter = 0.2 versus 1.2 ms), and better temporal modulation sensitivity (panel d, 222 versus 72 Hz) when compared to their paired ICNs. In contrast, ICNs have substantially narrower bandwidth than PBIs (panel e, 0.33 versus 0.94 octave) and prefer finer spectral modulations (panel f, 1.1 versus 0.2 cycle/octave). Open circle denotes broadband PBI, and triangle denotes narrowband PBI. Pairs recorded from the same tetrode are shown in black, and pairs recorded from adjacent tetrodes are shown in gray. Latency (panel a) and jitter (panel c) data are the same as Fig 2E and 2F, except that data are now grouped for broadband and narrowband PBI as well as same and adjacent tetrode pairs. Underlying data can be found in S5 Data and S5 Code. BB, broadband; ICN, inferior colliculus neuron; NB, narrowband; PBI, putative brainstem input.

A loss of response timing precision as you ascend the auditory pathways has been demonstrated with several experimental approaches [36]. If PBIs represent inputs to IC, we expect a reduction in temporal resolution between PBIs and ICNs. Indeed, ICNs always had longer sound-evoked latencies (100% of pairs, Fig 5A; median 8.6 versus 4.9 ms; Wilcoxon rank-sum test, *p* < 0.001) and longer integration times (Fig 5B, median 4.3 versus 2.3 ms; Wilcoxon rank-sum test, *p* < 0.001) than their paired PBIs. Spike-timing jitter for PBIs was roughly an order of magnitude smaller than that of paired ICNs, indicating more precise temporal responses (Fig 5C, median 0.23 versus 1.2 ms; Wilcoxon rank-sum test, *p* < 0.001). Furthermore, the cTMFs of the PBIs were substantially higher than those of ICNs, indicating phase-locked responses to faster features of the DMR sound (Fig 5D; median 222 versus 72 Hz; Wilcoxon rank-sum test, *p* < 0.001). Thus, temporal response properties of ICNs are characterized by slower processing and reduced temporal precision compared to the neighboring PBI.

In contrast to temporal selectivity, spectral selectivity was substantially better for ICNs. As evident from the population-average PBI and ICN STRF (Fig 4E–4H), ICNs have substantially narrower bandwidths (Fig 5E, median 0.33 versus 0.94 octave, Wilcoxon rank-sum test, *p* < 0.001). Furthermore, the cSMF of the ICNs are substantially higher than that of PBIs, indicating that ICNs can resolve substantially finer spectral details than their paired PBIs (Fig 5F, median 1.1 versus 0.2 cycle/octave, Wilcoxon rank-sum test, *p* < 0.001).

### Enhancing spectral resolution through degraded spike timing

Given the distinct differences between PBI and ICN receptive fields, we ask how the sharp tuning observed in ICNs could originate from a spectrally broad input receptive field. A plausible hypothesis is that sideband inhibition or cotuning between excitation and inhibition could enhance spectral selectivity for ICNs [37, 38]. Although there is substantial long-range inhibition from the brainstem to the IC [39] that could serve to sharpen tuning, there was no visible evidence for isolated inhibition between the PBI-ICN pairs because spike train crosscorrelograms with an isolated reduction in correlated firing were not observed.

**Fig 6:**
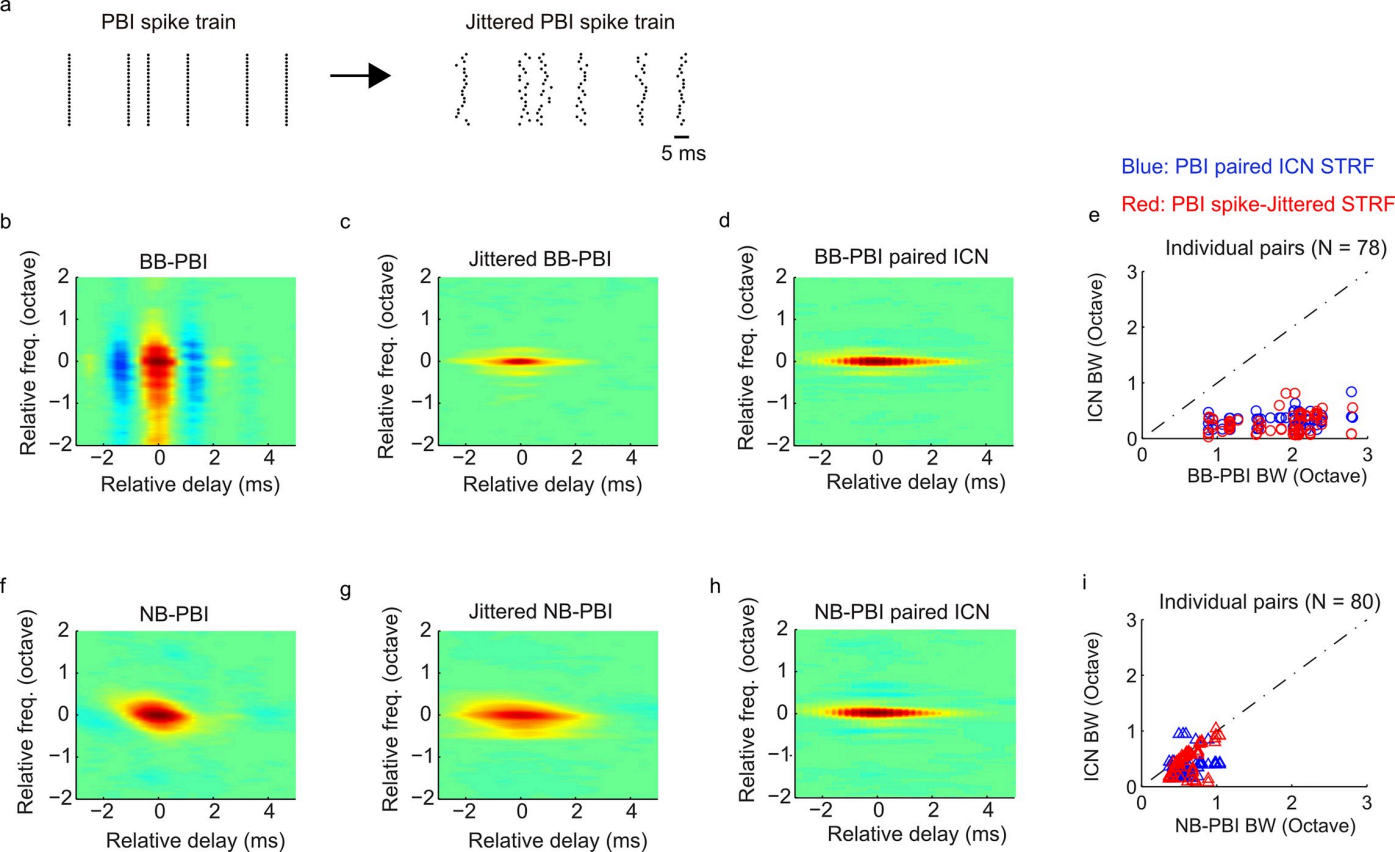
Reduced spike-timing precision enhances frequency selectivity of broadband PBIs but not for narrowband PBIs. (a) Synthetic spike-timing jitter (1.5 ms) was added to each of the PBI spike trains, and the STRF was recomputed. Although the population-average broadband PBI is broadly tuned (b), the jittered broadband PBI is substantially narrower (c) and closely matches the population-average ICN STRF at those sites (d). This enhanced frequency selectivity was evident after adding spike-timing jitter for each of the individual broadband PBIs (panel e, red dots: broadband PBI versus jittered broadband PBI bandwidth) and closely matched the observed measurements for individual paired broadband PBIs and ICNs (panel e, blue dots: broadband PBI versus ICN bandwidth). This behavior was not evident for narrowband PBIs (f–i) because the population-average STRF bandwidth was similar before (f) and after (g) adding spike-timing jitter. Furthermore, bandwidths before and after adding spike-timing jitter for individual narrowband PBIs (i) were significantly correlated (r = 0.6 ± 0.12), implying that the bandwidth after adding spike-timing jitter was largely inherited from the original narrowband PBI. Underlying data can be found in S6 Data and S6 Code. BB, broadband; ICN, inferior colliculus neuron; NB, narrowband; PBI, putative brainstem input; STRF, spectrotemporal receptive field.

An alternative although nontrivial possibility is that spike-timing differences between ICNs and PBIs could serve to enhance spectral selectivity observed for ICNs. As demonstrated in Fig 2, PBI and ICN spike trains were often temporally correlated although the spike-timing precision was substantially lower for ICNs, indicating a slower and less precise response. We thus asked whether temporal precision contributes to spectral selectivity and whether PBI activity with reduced spike-timing precision could predict ICN activity. To test this, we synthetically added spike-timing jitter to the original PBI spike trains and used these modified spike trains to re-estimate the STRFs (Fig 6A). The amount of jitter added to the PBI spike trains was chosen to be comparable to the measured jitter for ICNs, which had a median value of 1.2 ms (Fig 2F). Surprisingly, when we added spike-timing jitter (Gaussian distributed with 1.5 ms SD) to the original PBI spike trains, a substantial enhancement in spectral selectivity was observed for the jittered broadband PBI STRF (Fig 6B and 6C). Despite the broad structure of the broadband PBI (Fig 6B, population-average STRF shown), the resulting STRF obtained after adding jitter to the broadband PBI spike times (Fig 6C, population-average STRF shown) was substantially narrower and closely resembled the average ICN STRF (Fig 6D, population-average ICN STRF). This result was also replicated for each individual broadband PBI. The measured bandwidths were substantially narrower after adding spike-timing jitter (Fig 6E, red dots) and predicted the original bandwidth trend for paired broadband PBI and ICNs (Fig 6E, blue dots). This effect was not observed for the narrowband PBIs (Fig 6F–6I). Rather, the average narrowband PBI had comparable bandwidths before and after adding spike-timing errors (Fig 6F and 6G) such that the measured bandwidths before and after adding spike-timing jitter were highly correlated for individual narrowband PBI (Fig 6I, red dots, r = 0.6 ± 0.12). A somewhat weaker correlation was observed between the bandwidths of the corresponding paired narrowband PBI and ICN (Fig 6I, blue dots, r = 0.29 ± 0.09). This implies that bandwidths for this group of ICN were minimally transformed and partly inherited from the narrowband PBI. Thus, whereas ICN bandwidths from narrowband PBI sites appear to be partly inherited from the converging inputs, ICN bandwidths from broadband PBI sites are dramatically reduced, and these differences were accounted for by a reduction of timing precision of the broadly tuned inputs.

### Temporal integration mechanism for enhancing frequency selectivity

**Fig 7:**
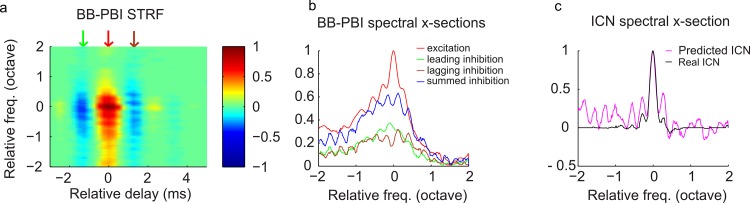
Constructive and destructive interference through integration of broadband PBI explains frequency sharpening of ICNs. (a) Population-average broadband PBI STRF has leading (green arrow) and lagging (brown arrow) inhibition around a central broadband excitatory domain (red arrow). (b) The spectral cross-sections are shown for the excitatory domain (red) along with the leading (green) and lagging (brown) inhibition and the summed inhibition (blue, leading + lagging component). The excitation has a sharp central peak around 0 octave frequency and adjacent peaks at intervals of approximately 0.28 octave. The summed inhibition has a broad profile with peaks that are spectrally displaced (out of phase) with the excitatory peaks. Upon summing the excitation and inhibition, the broad spectral structure cancels, and the narrowly tuned central region is preserved (panel c, magenta), which predicts the sharpened frequency tuning observed for ICNs (panel c, black, population-average ICN spectral cross-section). Underlying data can be found in S7 Data and S7 Code. BB, broadband; ICN, inferior colliculus neuron; PBI, putative brainstem input; STRF, spectrotemporal receptive field.

The idea that reduced timing precision can substantially sharpen spectral selectivity at broadband PBI sites is highly surprising. Although temporal integration from cell membrane properties can account for reduced timing precision in the IC [30, 36], we speculate that the spectral structure of the broadband PBI receptive fields plays a major role in the observed spectral sharpening, because a comparable phenomenon was not predicted for the narrowband PBI. The spectral and temporal structure of the average broadband PBI receptive fields provides some clues as to how sharpening of tuning might be achieved. These broadband PBIs contain interleaved excitatory and inhibitory domains at multiple delays (Fig 7A) with spectral peaks and notches that resemble a comb filter (multiple spectral peaks, Fig 7B). The spectral cross-sections of the average broadband PBI STRF are shown in Fig 7B at temporal delays corresponding to the leading (−1.25 ms, green arrow in panel a) and lagging (1.25 ms, brown arrow in panel a) inhibition and the central excitatory peak (0 ms, red arrow in panel a). Although the excitation and inhibition were overall broadly tuned, close inspection reveals the presence of interleaved peaks in the excitation and lagging inhibition with a spacing of approximately 0.28 octave. We tested whether constructive and destructive interference of these temporally delayed spectral components could sharpen spectral tuning. The summed inhibition (leading + lagging component, Fig 7B, blue) has a broad profile similar to the excitation (Fig 7B, red), but its interleaved peaks are precisely out of phase with the excitatory peaks. When we linearly subtracted the summed inhibition from the excitation, the broad spectral profiles cancel (Fig 7C, magenta), resulting in a spectral receptive field that is substantially narrower and accurately predicts the sharp ICN tuning around the BF (Fig 7C, ICN, black).

**Fig 8:**
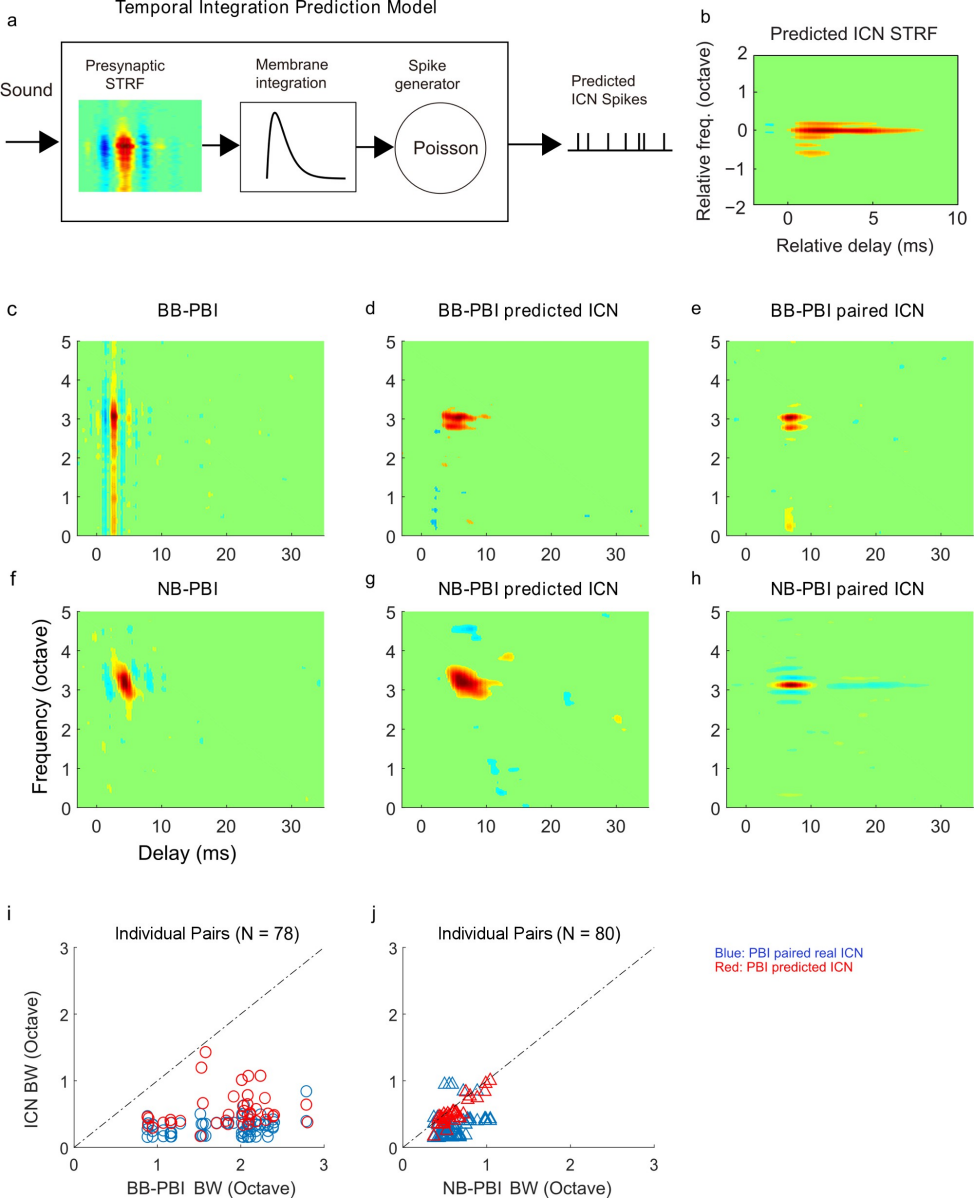
Temporal integration mechanism for sharpening frequency selectivity in ICNs. (a) A neuron model was developed to test the hypothesis that frequency selectivity can be sharpened through temporal integration of the converging broadband PBI. The model includes the PBI receptive field to account for presynaptic integration, a cell membrane integration compartment, and a Poisson spike train generator. When applied to the average broadband PBI STRF, the resulting STRF from the model spike train output sharpens dramatically and has similar spectral tuning and temporal profile (panel b) as the population-average ICN STRF (Fig 4F). (c–e) The model simulation is shown for an example broadband PBI site. The BB-PBI STRF has broad structure (panel c), whereas the predicted ICN STRF has narrowband structure (panel d) that closely mirrors the recorded ICNs (panel e). (f–h) The model simulation does not predict frequency sharpening for an NB-PBI site. The predicted ICN STRF has elongated temporal structure compared to the NB-PBI (panels f and g). However, the predicted STRF bandwidth is substantially larger than the corresponding ICNs (panel h) and comparable to that of the original PBI (panel f). Analogous to adding spike-timing jitter to individual PBI recordings (Fig 6E and 6I), the temporal integration model likewise replicates that paired bandwidth relationship for PBI and ICNs at individual sites (panels i and j). For BB-PBI sites, the model outputs have narrower bandwidths than the corresponding BB-PBI following a similar relationship to the original recordings (panel i). For NB-PBI sites, similar sharpening in frequency tuning for the model output or the recorded ICN is not observed (panel j). The measured bandwidths before and after the temporal integration model for individual narrowband PBIs were significantly correlated (r = 0.89 ± 0.03, mean ± SE; *p* < 0.01), implying that the bandwidth after adding spike-timing jitter was inherited from the original narrowband PBI. Underlying data can be found in S8 Data and S8 Code. BB, broadband; ICN, inferior colliculus neuron; NB, narrowband; PBI, putative brainstem input; STRF, spectrotemporal receptive field.

To test whether such constructive and destructive interference could be achieved through temporal integration of the cell membrane and nonlinear thresholding, we constructed an ICN model that contains a broadband PBI receptive field as its primary input (Fig 8A). The model consists of a broadband PBI receptive field to account for the presynaptic integration, a temporal integration kernel that accounts for the ICN cell membrane integration, and a stochastic Poisson spike-generating compartment to account for the nonlinear transformation between intracellular activity and extracellular spikes [29]. When the model kernel half-width is matched to the reported average integration time for the IC (5 ms) [30], the ICN model receptive field is substantially more narrowly tuned than its input, with an elongated temporal structure that resembles the ICN receptive field (Fig 8B). When the model was simulated for individual PBI sites, the broadband PBI showed a clear sharpening of the predicted ICN receptive field and similar structure as the recorded ICN (Fig 8C–8E). However, similar sharpening was not predicted when the simulation was performed at a narrowband PBI site (Fig 8F–8H). Statistics from simulating the model for individual same-tetrode PBI sites confirms these general trends (panels i and j). The model is able to predict the observed frequency sharpening for individual broadband PBI sites (panel i). However, a similar sharpening of ICN tuning is not predicted for the narrowband PBI sites (panel j). For both the model simulation (red dots, panel j) and neural data (blue dots, panel j), ICN bandwidths at the narrowband sites are correlated with the PBI bandwidths (blue dots, r = 0.29 ± 0.09; red dots, r = 0.89 ± 0.03; mean ± SE; *p* < 0.01), indicating that bandwidths at these sites are partly inherited from the PBI. Thus, the tuning characteristics of ICN receptive can be predicted from the neural response of the PBIs. ICNs at broadband PBI sites exhibit a dramatic enhancement in frequency selectivity, whereas a similar transformation is lacking at narrowband PBI sites. The result suggests that a simple temporal integration with a stochastic spike-generating mechanism and thresholding can account for the transformation of broadly tuned brainstem inputs to sharply tuned ICNs.

## Discussion

To understand how sound information is transformed from one neural structure to another, experimentalists often record neural activity simultaneously from both structures and look for synaptically connected neurons [5–7, 40, 41]. Here, we provide evidence that PPs can be recorded concurrently with postsynaptic action potentials and used to identify local transformations in the IC. These PPs have physiologic properties consistent with presynaptic potentials as previously observed in the giant synapse of squid [16], the endbulbs of held in the anterior ventral cochlear nucleus [2, 42], and the calyx of held in the medial nucleus of the trapezoid body [1, 2, 13, 15].

Our results provide evidence that (i) the recorded PPs are consistent with arising from brainstem input to the IC. (ii) These sources precisely target 1 or 2 neighboring frequency laminae and (iii) are temporally more precise but (iv) have poorer frequency resolution than the target ICN. (v) Finally, a temporal integration mechanism accurately accounts for the frequency sharpening observed in the neighboring ICN. This mechanism does not require local inhibition, thus providing a new temporal-based mechanism for enhancing sensory selectivity.

### Possible input sources to ICNs

Several physiologic and waveform characteristics of the identified PPs indicate that PBIs may represent presynaptic input to the IC. PBIs exhibited a highly consistent latency relationship and tight correlated firing between locally identified ICN. PBIs were also closely matched in frequency to ICNs with a resolution of approximately 0.3 octaves, as expected for converging inputs onto IC lamina with reported similar frequency resolution [32]. PBIs phase locked to faster sound modulations than neighboring ICNs, as would be expected for IC inputs. They consistently exhibited inverted waveform polarity (relative to ICNs), longer duration than recorded action potentials, and highly consistent waveforms across all four tetrode channels (small waveform disparity)—all properties that are expected for presynaptic potentials [27]. Although the small waveform disparity might be expected if the recorded potentials arise from distant neurons, the consistent latency relationship and close BF relationship with ICNs are both inconsistent with this idea. Furthermore, several physiologic (consistent short latency, lack of sound level relationship, measured interevent refractory period) and waveform (inverted polarity, fast waveform) properties are all inconsistent with the possibility that the PPs represent postsynaptic activity, including failed action potentials and summed synaptic potentials. The lack of sound level dependence and clear interevent refractory period are also inconsistent with summed afferent fiber potentials. Thus, the data support the general hypothesis that PBIs are input sources to the IC. Although we cannot fully confirm that PBIs are isolated input neurons, their low firing rate and well-identified interevent refractory period suggest that they likely represent input from a few brainstem sources.

It is unlikely that the recorded PBIs project broadly to ICNs since they were observed in only 19% of the recording locations. Instead, PBIs may correspond to a distinct subset of inputs to specific neural types in the IC. One candidate cell type with morphological properties that could produce strong PPs as identified here is the large GABA projecting neuron with calyx-like synapses that have been recently identified in the IC [28]. The inputs to these neurons are suspected to originate in the dorsal cochlear nucleus and/or superior olivary complex [43]. Unlike the dominant disk-shaped cell present throughout the IC, these cells have large somas, and a high density of excitatory axosomatic terminals may be capable of generating PPs as described here. An argument against this possibility is that the recorded PBI and ICN waveforms should co-locate spatially since both waveforms would originate on the soma and one would expect similar recording radius for both signals, which we did not observe. A plausible alternately is that the PP signals arise from converging input to the dendritic terminals of ICNs. This possibility is supported by the fact that PBIs have a larger recording radius spanning approximately 500 um and the fact that dendritic terminal in ICNs typically span 250 to 500 μm with an upper limit of approximately 1,000 μm in cat [34]. Furthermore, the dominant disk-shaped cells in the IC have dendrites confined to iso-frequency lamina spanning approximately 1/3 octave in cat [32] and roughly 150 μm in their short dimension [34], consistent with the tight frequency difference distribution between PBI and ICN. However, these disk-shaped cell dendrites can extend up to approximately 1,000 μm on their long dimension, which is consistent with a large radius for generating potentials.

Two distinct groups of PBIs were identified with functionally different properties that may reflect inputs from anatomically distinct brainstem pathways. Broadband PBIs are faster and more broadly tuned then the slower narrowband PBIs. Because of their short latency, fast temporal properties, broad bandwidths, and the fact that they are strictly monaural (100% broadband PBIs did not have an ipsilateral STRF), we speculate that broadband PBI could originate from the dorsal cochlear nucleus principal cell inputs to the IC. Although monaural responses on their own are not sufficient to implicate the dorsal cochlear nucleus [44, 45], the broadband on-off temporal receptive field structure with multiple spectral peaks of the broadband PBIs closely resembles those previously observed for dorsal cochlear nucleus STRFs [46, 47]. Thus, based on the receptive field similarity, the dorsal cochlear nucleus could be a likely source of input. By comparison, narrowband PBIs are slower, have narrower bandwidths, and can exhibit binaural properties; 13% had statistically significant contralateral and ipsilateral STRFs (*p* < 0.001), suggesting that they could potentially originate in the superior olivary complex.

### Functional transformation between putative inputs and ICNs

The findings suggest that the recorded PBIs are precisely arranged to target 1 or 2 adjacent laminae and that their bandwidths can be refined to critical band-like resolution in the brainstem to IC transformation. Although prior studies had identified approximately 1/3 octave output organization for ICN responses [31, 32], our results support a bottom-up organization whereby the inputs themselves are organized to target 1/3 octave laminar intervals. The frequency range for the most strongly correlated PBI-ICN activity falls within the limits of a single critical band, the standard for frequency resolution in humans and other mammals [17, 18]. Adjacent laminae are approximately 150 μm apart [33, 34] and are separated in frequency by about 0.28 octave [32] in the cat. This closely matches the spacing between peaks in the BF difference distribution for PBI and ICN pairs (Fig 4), suggesting that PBI-ICNs pairs arise from neighboring sources typically in the same frequency band laminae (same-tetrode pairs) and at most 2 laminae apart (for adjacent-tetrode pairs). This is also consistent with our estimates of the PBI recording radius (about 450 μm median), which spans the bounds of just a few lamina. Finally, PBIs were most effective at generating ICN spikes and contributed most when paired PBIs and ICNs were closely matched in frequency and within the bounds of 1 or 2 fibrodendritic laminae (clusters at 0 and 0.26 octave BF difference, Fig 3). The low observed contribution suggests that ICN responses are not dominated by a single input. This is consistent with IC anatomy, since brainstem inputs are organized into functional zones that receive inputs from multiple sources that project onto ICNs allowing for merging and generation of new response selectivities [9, 39, 48].

Dramatic differences in frequency tuning as described here have not been observed between the auditory nerve, brainstem, and IC with conventional tone-based response areas [49, 50]. Although we delivered search tones at multiple frequencies and sound pressure levels for some recording sites, the cluster analysis did not identify any PPs under such conditions. One key difference between conventional tone-based measures and the measures of frequency selectivity used here is that the STRFs measure temporally phase-locked neural activity to the sound modulations down to sub-millisecond time scales. By comparison, tone-based measures of frequency selectivity only measure average firing rates to relatively longer sustained sounds (i.e., about 50–100 ms). Thus, our results reflect frequency selectivity for brief acoustic events that is reflected in precise timing of neural responses. Another factor is that the receptive fields of PBIs exhibited very broad tuning and a fast on-off-on temporal arrangement, which would require a brief (a few milliseconds) and highly synchronous broad input to effectively drive them. Such broad and temporally fast acoustic features are evident in the dynamic ripple sounds employed here, yet they are uncharacteristic of tones. A final relevant point is that although frequency bandwidths can be quite heterogeneous, prior studies comparing peripheral and IC bandwidths did so by computing population averages [49, 50]. Here, we compared PBIs and ICNs in close proximity enabling comparisons on a neuron-to-neuron basis and thus allowing us to identify local transformations that cannot be identified by comparing population averages.

Modulation preferences also undergo a major transformation between PBIs and ICNs (Figs 4 and 5) that are consistent with reported changes in modulation selectivity between brainstem and IC [36]. PBIs can phase-lock to substantially higher temporal modulations than ICNs; however, ICNs have substantially better spectral modulation sensitivity and thus can respond selectively to finer details of the sound spectrum. Although spectral selectivity and the observed upper limits of temporal modulation sensitivity may be somewhat affected by the use of anesthesia, the reduced levels of neural activity may be beneficial and may have contributed to our ability to identify PBI. Anesthesia lowers spike rates so that neurons are activated more sparsely, which should improve the detection quality of the spike sorter.

Despite dramatic differences between broadband and narrowband PBI, the recorded ICNs for each site seem to produce similar average receptive fields. Although there are some subtle differences in the inhibitory components of ICNs, the main excitatory component is remarkably similar for the average broadband and narrowband PBIs (Fig 4G and 4H). This apparent homogenization of the ICN responses is somewhat surprising, because one would expect that some of the receptive field properties would be inherited from the converging input. However, it is worth noting that individual ICN bandwidths at narrowband sites are significantly correlated with their PBI bandwidths (Figs 6I and 8J), indicating that the ICN bandwidths for narrowband sites are partly inherited from the PBI. Furthermore, ICNs at narrowband sites exhibited weak but significant sideband inhibition and consequently preferred finer detailed spectral modulation (higher cSMF) than ICNs at narrowband sites. A factor to consider when interpreting these data is that nonlinearities shape the PBI-ICN transformation and that the STRF only captures a subset of the phase-locked acoustic features that ICNs respond to [20, 51, 52]. For instance, it is possible that ICNs at broadband sites are biased to respond to broad transient sound elements, whereas ICNs at narrowband sites would be less selective for such structure. Yet for either case, the phase-locked activity of the spiking output might synchronize only to a narrow frequency band about the neuron’s BF, which would be reflected in the linear projection component (i.e., the STRF). Such nonlinear transformation is plausible, since ICNs can show multiple selectivities, including nonlinear or non–phase-locked response components, which don’t show up in the STRF and often differ from the linear components [20, 51, 52]. Functionally, differences between broadband and narrowband PBIs may be relevant for detecting transient sound elements and for detecting correlations between frequency channels, which are both relevant perceptually and for neural coding in the IC [20, 53, 54].

Perhaps the most intriguing result is that frequency selectivity can be quickly focused from broadly tuned inputs, which is accurately explained by a fast temporal integration mechanism. This finding differs from classic models for enhancing frequency selectivity, which rely on local inhibition as a means of sculpting frequency selectivity [13, 37, 38, 42]. Although local inhibition is important for shaping sound preferences in the IC [11, 37], these results provide a surprising alternative mechanism for enhancing frequency selectivity. Our results indicate that reduced timing precision of broadband PBI spike trains alone is sufficient to account for the narrowly tuned structure of the recorded neighboring ICNs. Our model provides further confirmation for how temporal integration of broadly tuned inputs can quickly refine the frequency tuning (Fig 8). The receptive fields of broadband PBIs have temporally interleaved excitation and inhibition (Fig 7) with spectral peaks that are out of phase. This arrangement of the converging input is precisely balanced so that temporal integration and nonlinear thresholding in the recipient ICN can cancel off-BF inputs, thus sharpening frequency tuning. This enhancement in tuning can be achieved quickly, within a few milliseconds, thus allowing for fast signaling of fine frequency details within the time course of a single action potential.

## Materials and methods

### Ethics statement

Animals were handled according to approved procedures by the University of Connecticut Animal Care and Use Committee (Protocol A07-036) and in accordance with NIH and AVMA guidelines.

### Surgical procedure

Surgical and experimental procedures have been reported in detail elsewhere [19, 35] and are briefly outlined here. Adult cats (*Felis catus*, *N* = 6) were anesthetized with a mixture of ketamine (10 mg/kg) and acepromazine (0.28 mg/kg I.M.) and were subsequently maintained in a surgical state with either sodium pentobarbital (30 mg/kg, *N* = 2) or isoflurane gas mixture (3%–4%, *N* = 4). The IC was exposed by removing the overlying cortical tissue and the bony tentorium. Following surgery, the animal was maintained in a reflexive state by continuous infusion of ketamine (2 mg/kg·h) and diazepam (3 mg/kg·h) in a lactated ringers solution (4 mg/kg·h). Physiologic data (heart rate, temperature, breathing rate, and reflexes) were monitored to control the infusion rate.

### Acoustic stimuli and delivery

Sounds were delivered in a sound-shielded chamber (IAC, Bronx, NY) via hollow ear-bars (Kopf Instruments, Tujunga, CA) using calibrated speaker drivers (200 Hz–40 kHz, ±3 dB; Beyer DT770). Sounds were delivered with either a TDT RX6 (Alchua, FL) or an RME DIGI 9652 (Haimhausen, Germany).

We first delivered broadband noise and tone pips (1–47 kHz) to identify single units and to verify the tonotopic gradient of the ICC [55]. Next, a DMR sound was delivered dichotically to measure the spectrotemporal preferences [20, 35]. The DMR is a time-varying broadband sound (1–48 kHz; 96 kHz sampling rate) that contains spectral (0–4 cycles/octave) and temporal (0–500 Hz) modulations that have been shown to efficiently activate ICC neurons and are prominent features in natural sounds [20, 35]. A 10-min sequence of the DMR was presented twice (Trial A and Trial B, 20 min total) at fixed intensity (80 dB SPL, 65 dB spectrum level per 1/3 octave).

### Electrophysiology

Acute 4-tetrode (16-channel) recording probes (2 shanks with 2 tetrode sites on each, 177 μm^2^ contact area with impedance 1.5–3.5 MΩ at 1 kHz; NeuroNexus Technologies, Ann Arbor, MI) were used to record neuronal activity from the ICC. The interelectrode distance within a tetrode is 25 μm, and the distance between adjacent tetrode sites is 150 μm. The probes were advanced stereotaxically (Kopf Instruments, Tujunga, CA) at an angle of approximately 30° relative to the sagittal plane (orthogonal to the frequency-band lamina) [32] using an LSS 6000 Inchworm microdrive (Burleigh EXFO; Vanier, Quebec). Efforts were made to sample different regions of the ICC by moving the electrode along the mediolateral and rostralcaudal axis. At each penetration location, we advanced the probe depth up to 3 mm and recorded only from locations that followed a clear tonotopic gradient [55]. Best frequencies with this recording strategy were confined to the range of 1.3 to 16.7 kHz (median 6.5 kHz).

### Identifying PBIs via waveform cluster analysis

Neural responses were digitized and recorded with an RX5 Pentusa Base station (TDT, Alchua, FL) followed by offline analysis in MATLAB (MathWorks, Natick, MA). The continuous neural traces were digitally band-pass filtered (300–5,000 Hz), and cross-channel covariance was computed across tetrode channels [56]. Vectors consisting of the instantaneous channel voltages across the tetrode array exceeded a hyperellipsoidal threshold *V*^*T*^*C*^−1^*V*>*f*^2^, where *V* is the vector of voltages, *C* is the covariance matrix, and *f =* 5 is the normalized threshold level [56]. Because the channels are partly correlated, and the covariance has nondiagonalized structure, the average composite signal measured *V*^*T*^*C*^−1^*V* had a normalized power of 2.1 units. Thus, the equivalent threshold level at which the spike sorter detected events was approximately 3.5 SD ($\sqrt{f^{2}/2.1}=3.5$). Detected waveforms (action potentials or PPs) were aligned, and 1.5 ms length of snippets was used for spike sorting using KlustaKwik software [23]. Three parameters were used for spike sorting: positive peak value, negative peak value, and first-order principle component. Clusters were kept only if the interspike intervals exceeded 0.5 ms for >95.5% of the detected events and the signal-to-noise ratio exceeded 2 (6 dB).

Waveforms for each identified neural event (spikes or PPs) were extracted, and waveform statistics were used to blindly segregate ICN and PBI. This procedure ensured that PBIs and ICNs were identified independently of each other by strictly employing waveform criteria. There was no visual identification or user intervention in this process. The waveform width was defined by the peak-to-peak width of the waveform with the biggest amplitude among the 4 tetrode channels. The waveform frequency was defined by the peak frequency of the Fourier transform of the same signal. The interchannel disparity was defined by the normalized mean square error of the waveforms across 4 channels. Euclidean distance minimum variance cluster analysis algorithm was then applied to the 3 parameters (Fig 1F: peak-to-peak width, peak waveform frequency, and interchannel disparity). The cluster analysis resulted in 103 and 463 independently identified PBIs and ICNs, respectively. We identified 37 PBI-ICN pairs that were recorded concurrently on the same tetrode and 121 PBI and ICN pairs that were recorded concurrently on adjacent tetrodes (150 μm apart).

### Spectrotemporal analysis

STRFs for the contralateral ear of identified ICC single neurons were obtained using spike-triggered averaging [20], and spectrotemporal parameters were obtained for each STRF according to the procedure described previously [35]. Briefly, latency and BF were defined by the peak of the STRF power marginal. Integration time and bandwidth were defined as twice the SD of the STRF power marginals. Excitatory (+) and inhibitory (−) samples of the STRFs were each tested at a significance level of *p* < 0.001 as outlined previously [20]. Throughout the manuscript, analysis is carried out only on statistically significant STRF subfields, and only the statistically significant samples used for analysis are shown in the figures.

For each unit, we derived spectral and temporal modulation response parameters directly from the ripple transfer function (RTF). The RTF of each unit is obtained by computing the two-dimensional Fourier transform of its STRF and subsequently computing the transfer function magnitude. The cTMF and cSMF parameters were obtained as the centroids from the modulation power marginal of the RTF (equations 8–9 in [35]).

### Spike-timing precision (jitter)

We measured the spike-timing precision of each neuron using a shuffled correlogram algorithm applied to responses from 2 trials of the DMR (Trial A and B). The SAC was computed as

$$\phi_{shuffled}(\tau )=\frac{\phi_{AB}(\tau )+\phi_{BA}(\tau )}{2}$$

where *ϕ*_*XY*_(*τ*) = 〈*s*_*X*_(*t*)*s*_*Y*_(*t*+*τ*)〉 is the crosscorrelogram between trial *X* and *Y*, *s*_*X*_(*t*) is the spike train for trial *X*, *s*_*Y*_(*t*) is the spike train for trial *Y*, and $\langle ∙\rangle =1/T\int_{0}^{T}∙dt$ is the time average operator.

The precision of firing was estimated by fitting the SAC to a Gaussian model of the form [57]

ϕmodel(τ)=λ2+pλ14πσ2e−τ24σ2

where *p* is the firing reliability, *λ* is the firing rate, and *σ* is the standard deviation of the spike-timing jitter. The parameter *σ* and *p* were obtained by numerically fitting the model to the experimentally measured shuffled correlogram using constrained least squares optimization where *λ* = *L*/*T*, *L* is the number of spikes, *T* is the spike train duration, *σ*>0, and 0≤*p*≤1.

### Spike train correlogram, contribution, and efficacy

For each PBI-ICN pair, we computed the crosscorrelogram [58] as a measure of the functional correlation between each PBI and ICN. Both the PBI and ICN spike trains were sampled at rate of 4 kHz, and crosscorrelograms were computed with maximum delay of 25 ms. Significance of correlation was tested on a chance level of *p* < 0.001 compared with bootstrapped crosscorrelograms after randomizing the spike timing of each spike train by shuffling the interspike intervals. Functional connection strength was quantified as efficacy (area underneath the correlogram exceeding baseline from −2 to 2 ms around the peak, normalized by the presynaptic spike rate) and contribution (the same quantity but normalized by the postsynaptic spike rate). Thus, efficacy is the percentage of PBI spikes that are followed by an ICN spike, and contribution is the percentage of ICN spikes that are preceded by a PBI spike.

For each unit, we also estimated the refractory period between consecutive spikes using correlogram analysis. The refractory period was defined by a lack of correlated firing in the autocorrelogram within the vicinity of a 0 ms delay [23, 59, 60]. Within this framework, the refractory period does not necessarily represent sodium channel inactivation. Instead, it refers to the absence of spikes in the post-spike activity, which may arise through a variety of mechanisms (including sodium channel inactivation, nonlinear cell-membrane integration and thresholding, etc.).

## Supporting information

S1 Data MATLAB formatted file containing, in a data structure “Fig 1,” the numerical data values for Fig 1, panels c, e, f, and g.
(MAT)

S2 Data MATLAB formatted file containing, in a data structure “Fig 2,” the numerical data values for Fig 2, panels a-f.
(MAT)

S3 Data MATLAB formatted file containing, in a data structure “Fig 3,” the numerical data values for Fig 3, panels a-f.
(MAT)

S4 Data MATLAB formatted file containing, in a data structure “Fig 4,” the numerical data values for Fig 4, panels a-h.
(MAT)

S5 Data MATLAB formatted file containing, in a data structure “Fig 5,” the numerical data values for Fig 5, panels a-f.
(MAT)

S6 Data MATLAB formatted file containing, in a data structure “Fig 6,” the numerical data values for Fig 6, panels b-i.
(MAT)

S7 Data MATLAB formatted file containing, in a data structure “Fig 7,” the numerical data values for Fig 7, panels a-c.
(MAT)

S8 Data MATLAB formatted file containing, in a data structure “Fig 8,” the numerical data values for Fig 8, panels b-j.
(MAT)

S1 Code MATLAB code to read S1 Data.mat and generate Fig 1 panels. (M)

S2 Code MATLAB code to read S2 Data.mat and generate Fig 2 panels. (M)

S3 Code MATLAB code to read S3 Data.mat and generate Fig 3 panels. (M)

S4 Code MATLAB code to read S4 Data.mat and generate Fig 4 panels. (M)

S5 Code MATLAB code to read S5 Data.mat and generate Fig 5 panels. (M)

S6 Code MATLAB code to read S6 Data.mat and generate Fig 6 panels. (M)

S7 Code MATLAB code to read S7 Data.mat and generate Fig 7 panels. (M)

S8 Code MATLAB code to read S8 Data.mat and generate Fig 8 panels. (M)

## Abbreviations

BF: best frequency
cSMF: characteristic spectral modulation frequency
cTMF: characteristic temporal modulation frequency
DMR: Dynamic Moving Ripple
IC: inferior colliculus
ICC: central nucleus of the IC
ICN: IC neuron
LFP: local field potential
PBI: putative brainstem input
PP: prepotential
RTF: ripple transfer function
SAC: shuffled autocorrelogram
STRF: spectrotemporal receptive field
